# Analysis of potential biomarkers for diabetic kidney disease based on single-cell RNA-sequencing integrated with a single-cell sequencing assay for transposase-accessible chromatin

**DOI:** 10.18632/aging.205107

**Published:** 2023-10-11

**Authors:** Yan Shi, Zuishuang Guo, Fengxun Liu, Shaokang Pan, Dan Gao, Sijie Zhou, Zhenjie Liu, Feng Wang, Dongwei Liu, Zhangsuo Liu

**Affiliations:** 1 Traditional Chinese Medicine Integrated Department of Nephrology, The First Affiliated Hospital of Zhengzhou University, Zhengzhou University, Zhengzhou 450052, P.R. China; 2 Research Institute of Nephrology, Zhengzhou University, Zhengzhou 450052, P.R. China; 3 Henan Province Research Center for Kidney Disease, Zhengzhou 450052, P.R. China; 4 Key Laboratory of Precision Diagnosis and Treatment for Chronic Kidney Disease in Henan Province, Zhengzhou 450052, P.R. China; 5 Department of Nephrology, Shanghai Eighth People’s Hospital, Shanghai Jiao Tong University Affiliated Sixth People’s Hospital, Shanghai 200233, P.R. China

**Keywords:** scATAC-seq, scRNA-seq, db/db mice, diabetic kidney disease, renal tubule

## Abstract

Diabetic kidney disease (DKD) is a renal microvascular disease caused by hyperglycemia that involves metabolic remodeling, oxidative stress, inflammation, and other factors. The mechanism is complex and not fully unraveled. We performed an integrated single-cell sequencing assay for transposase-accessible chromatin (scATAC-seq) and single-cell RNA-sequencing (scRNA-seq) analyses of kidneys from db/db and db/m mice to identify differential open chromatin regions and gene expression, particularly in genes related to proximal tubular reabsorption and secretion. We identified 9,776 differentially expressed genes (DEGs) and 884 cell type-specific transcription factors (TFs) across 15 cell types. Glucose and lipid transporters, and TFs related to the circadian rhythm in the proximal tubules had significantly higher expression in db/db mice than in db/m mice (*P*<0.01). Crosstalk between podocytes and tubular cells in the proximal tubules was enhanced, and renal inflammation, oxidative stress, and fibrosis pathways were activated in db/db mice. Western blotting and immunohistochemical staining results showed that *Wfdc2* expression in the urine and kidneys of DKD patients was higher than that in non-diabetic kidney disease (NDKD) controls. The revealed landscape of chromatin accessibility and transcriptional profiles in db/db mice provide insights into the pathological mechanism of DKD.

## INTRODUCTION

The prevalence of diabetic kidney disease (DKD) is increasing annually, and it has been exacerbated by unhealthy lifestyles. Approximately 537 million people have been diagnosed with diabetes around the world in 2021, and this number is projected to reach 783 million by 2045 [1]. Patients with diabetes have a 50% increased risk of kidney damage, and DKD is the primary cause of end-stage renal disease in developed countries [2]. Early DKD diagnosis mainly relies on microalbuminuria testing; however, this test is not sufficiently sensitive or specific [3]. The onset of DKD is insidious and irreversible, and no effective biomarker or treatment has been identified. Therefore, early diagnosis and treatment of DKD remain challenging. Renal disease onset and development were traditionally considered to be mainly caused by the destruction of glomerular architecture or function. However, renal function has been found to be more strongly correlated with tubular function than with glomerular function in patients with chronic kidney disease [4]. Recently, the association between renal tubular function and DKD has attracted increasing research attention.

The physiological structure of the kidneys is highly complex, comprising more than 1 million nephrons, 14 renal tubule segments, and more than 40 different cell types [5]. This complexity and variety of cell types limit in-depth studies on kidney disease. However, the development of single-cell sequencing technology has facilitated the study of human disease at a single-cell resolution. The single-cell sequencing assay for transposase-accessible chromatin (scATAC-seq) is an innovative sequencing method for exploring chromatin accessibility, and integrative analysis of scATAC-seq and single-cell RNA-sequencing (scRNA-seq) data can be used to synchronously assess the regulation of gene input and output.

The C57BLKS/J-Lepr db mouse model (with a mutation in the leptin receptor) is widely used to study DKD. Homozygous C57BLKS/J-Lepr db mice (db/db) are infertile, exhibit a diabetes phenotype (hyperglycemia, hyperlipidemia, and insulin resistance), and develop proteinuria at 8–12 weeks of age. The pathogenesis of diabetes-like kidney disease in db/db mice is similar to that of type 2-DKD in humans. Heterozygous C57BLKS/J-Lepr db mice (db/m) show a normal phenotype [6] and are used as a control.

The renal tubular hypothesis has been proposed to explain the pathogenesis of DKD. This hypothesis describes the physiological and pathological mechanisms of the renal tubules responding to hyperglycemic stimulation, which subsequently affects the glomerular and tubular functions of the kidneys in patients with diabetes. Under hyperglycemic conditions, renal tubular reabsorption of glucose and sodium and passive reabsorption of chloride and water increase. The glomeruli undergo hyperfiltration in response to increased reabsorption by the renal tubules, which can manifest as an increase in the glomerular filtration rate (GFR) [7, 8]. Tubular cells undergo hypertrophy, function at maximum capacity, have an increased demand for energy and oxygen, and become extremely fragile under these conditions. The renal tubular hypothesis explains the early structural and functional changes observed in DKD, including increased tubular reabsorption; metabolic changes; and changes in growth, proliferation, oxidative stress, inflammation, and fibrosis of tubular epithelial cells [9–11]. However, the mechanism underlying the renal tubular hypothesis and the regulatory networks involved remain unclear. In this study, we performed combined scATAC-seq and scRNA-seq of kidney cells from 16-week-old male db/db and db/m mice with the aim to investigate early renal tubular functional and pathological changes in DKD.

## RESULTS

### Single-cell transcriptome analysis of the kidneys from db/db and db/m mice

**Figure 1:**
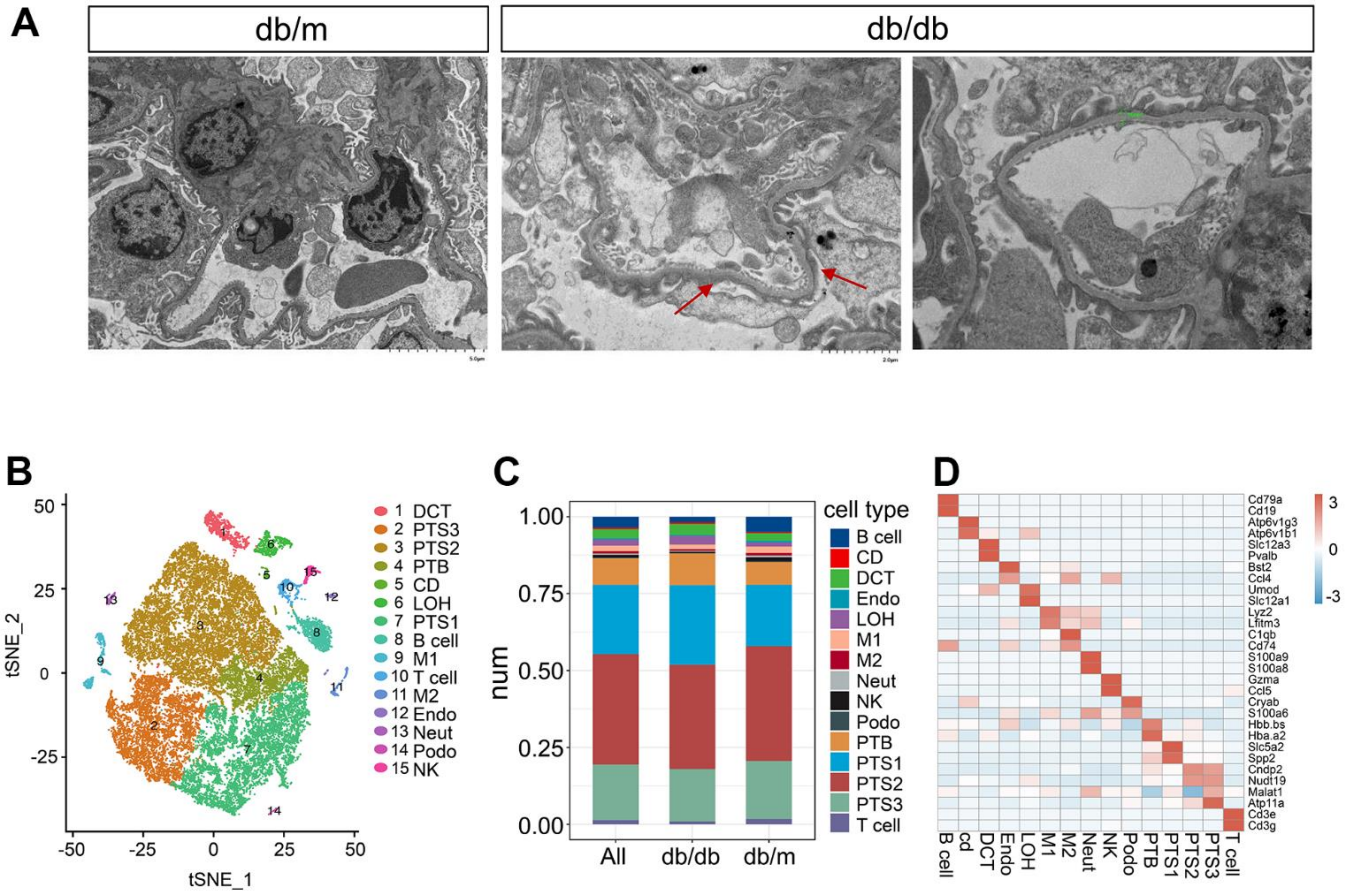
**Single-cell transcriptome analysis in db/db mice.** (**A**) Transmission electron micrographs of glomerulus from db/db and db/m mouse kidneys. The red arrow points towards the foot process fusion on the glomerular basement membrane. The green bar in the db/db image on the right side indicates the thickening of Glomerular basement membrane. (**B**) tSNE visualization of kidney cells from db/db and db/m mice, colored by cell type. DCT, distal convoluted tubule; PTS3, third segment of the proximal tubule; PTS2, second segment of the proximal tubule; PTB, proximal tubule brush; CD, collecting duct; LOH, loop of Henle; PTS1, first segment of the proximal tubule; B cell, B lymphocytes; M1, classical macrophage; T cell, T lymphocyte; M2, alternatively activated macrophage; Endo, endothelial cells; Neut, neutrophil progenitor; Podo, podocyte; NK, natural killer cell. (**C**) Bar plot showing the percentages of transcriptionally defined cell populations in db/db and db/m mice. The colors correspond to the cell types identified. (**D**) Heatmap of the mean expression of two manually selected marker genes in each cell type. Gene expression was standardized between –3 and 3 and is indicated by color intensity.

We performed scATAC-seq on kidneys of 16-week-old db/db and db/m mice to characterize the accessible chromatin landscape of kidneys stimulated by hyperglycemia. Transmission electron microscopy (TEM) revealed obvious structural changes in db/db mouse kidneys compared to those in db/m mouse kidneys, including increased accumulation of the extracellular matrix, thickened basement membrane, and fusion of foot processes. (Figure 1A). A total of 42,833 cells from six kidneys obtained from three db/db and three db/m mice were processed for scRNA-seq on the 10× Genomics Chromium platform, with an average of 57,099 reads per cell. After quality control, 32,058 cells were included in the analysis (Supplementary Table 1), comprising approximately 13,906 cells from db/db mice and 18,152 cells from db/m mice, with an average of 2,096 genes per cell.

A graph-based clustering method based on the standardized expression of marker genes (Supplementary Table 2) was used to annotate the following 15 distinct populations, which were visualized in the t-distributed stochastic neighbor embedding (tSNE) space (Figure 1B): the distal convoluted tubule (DCT; expressing *Slc12a3, Pvalb*), third segment of the proximal tubule (PTS3; expressing *Atp11a, Malat1*), second segment of the proximal tubule (PTS2; expressing *Cndp2, Nudt19*), proximal tubule brush (PTB; expressing *Hbb.bs, Hba.a2*), collecting duct (CD; expressing *Atp6v1g3, Atp6v1b1*), loop of Henle (LOH; expressing *Umod, Slc12a1*), first segment of the proximal tubule (PTS1; expressing *Slc5a2, Spp2*), B lymphocytes (B cells; expressing *Cd79a, Cd19*), T lymphocytes (T cells; expressing *Cd3e, Cd3g*), classical macrophages (M1; expressing *Lyz2, Cfh*), alternatively activated macrophages (M2; expressing *C1qb, Cd74*), endothelia (Endo; expressing *Bst2, Ccl4*), neutrophil progenitors (Neut; expressing *S100a9, S100a8*), podocytes (Podo; expressing *Cryab, S100a6*), and natural killer cells (NK; expressing *Gzma, Ccl5*). The transcriptional signatures of these 15 cell clusters were identified. The cell numbers in each cluster in db/db and db/m mice are listed in Supplementary Table 3, and their proportions are shown in Figure 1C. Proximal tubule cells were the most abundant in all clusters, accounting for approximately 85% of total cells, whereas podocytes were the least abundant, accounting for approximately 0.3% of cells, followed by collecting duct cells. Compared with db/m mice, db/db mice had significantly decreased cell numbers in the Podo, PTS3, PTS2, and Endo clusters (*P*<0.01) but significantly increased cell numbers in the LOH, CD, PTB, PTS1, and DCT clusters (*P*<0.01, Figure 1B and Supplementary Table 3). The numbers of M1, M2, T, B, and Neut cells significantly decreased in db/db mice (*P*<0.01).

The top two marker genes from each cell type ranked by significance after standardization are shown in Figure 1D. We analyzed the expression patterns of specific transporters in the renal tubules. Genes encoding aquaporin 11 (*Aqp11*) and transporters for glucose (*Slc2a2*), organic cations (*Slc22a1*), organic anions (*Slc22a8*), amino acids (*Slc3a1, Slc7a9, Slc1a1,* and *Slc6a18*), uric acid (*Slc22a12*), sulfate (*Slc13a1*), phosphate (*Slc34a1*), and thyroid hormone (*Slc16a2*) were specifically expressed in the proximal tubules (Supplementary Figure 1A–1C). The expression of gene encoding aquaporin 6 (*Aqp6*) was specific to the CD, the expression of genes encoding sodium and chloride iron transporter (*Slc12a3*) and serine/threonine-protein kinase (*Trpm6*) were specific to the DCT, the expression of gene encoding sodium, potassium and chloride ion transporter (*Slc12a1*) was specific to the LOH (Supplementary Figure 1D).

### Comparative analysis of renal tubule cells from db/db and db/m mice

**Figure 2:**
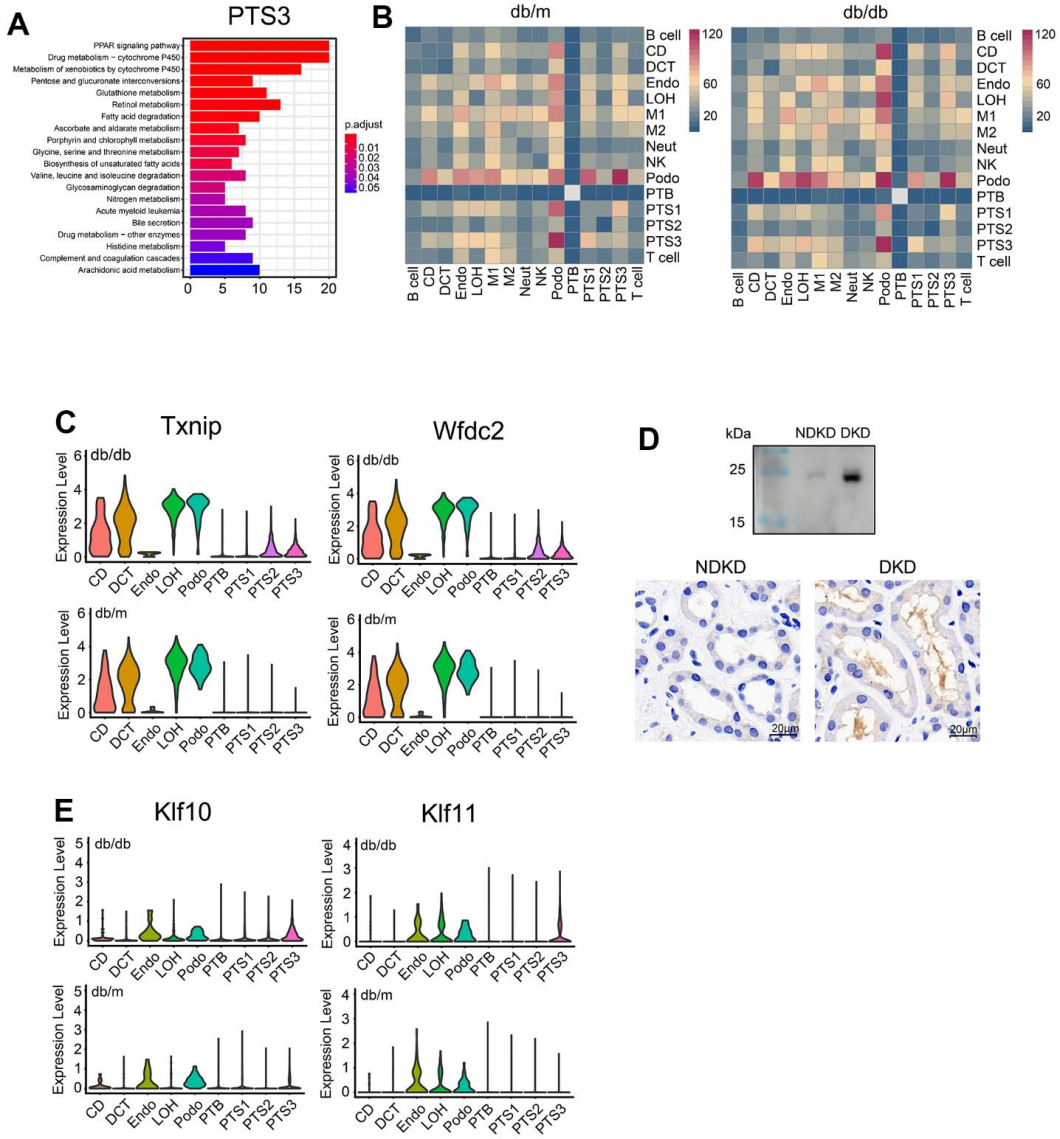
**Comparative analysis of differentially expressed genes between db/db and db/m mice.** (**A**) Functional enrichment analysis of the upregulated genes in the PTS3 cluster based on KEGG pathway analysis. The color corresponds to the significance values. (**B**) Heatmap showing the numbers of cell–cell interactions in the scRNA-seq datasets of db/m mice and db/db mice. The strength of cell–cell communication is indicated by color intensity. (**C**) Differential expression of *Txnip* and *Wfdc2* in db/db and db/m mice. (**D**) *Wfdc2* expression in the morning urine and kidneys of patients with diabetic kidney disease (DKD) and non-diabetic kidney disease (NDKD) controls, as analyzed by western blotting and immunohistochemistry. (**E**) Violin plots showing the expression of *Klf10* and *Klf11* in the kidneys of db/db and db/m mice.

PTS3 had the most differentially expressed genes (DEGs) between db/db and db/m mice (n = 6,284), followed by PTS1 and PTS2, whereas CD had the least DEGs (n = 140), followed by Neut (Supplementary Table 4). Kyoto Encyclopedia of Genes and Genomes (KEGG) analysis showed that the significantly upregulated genes in the PTS3 cluster of db/db mice were mainly involved in the PPAR signaling pathway and fatty acid absorption and degradation (Figure 2A). In the tubules, such as LOH, CD, and DCT, the significantly upregulated genes in db/db mice were mainly involved in the MAPK and TGF-β signaling pathways, whereas those in Podo and Endo cells were mainly involved in the Wnt and MAPK signaling pathways (Supplementary Figure 2).

Analysis of cell communication showed a significant enhancement in the kidneys of db/db mice, especially between podocytes and tubular cells (Figure 2B). Similarly, the analysis of intercellular receptor–ligand pairs revealed that the expression of ligand receptors for Sema4D-PlexnD1 and Sema4D-PlexnB1 was higher and that of ligand receptors for VEGFB_ADRB2 and VEGFB_NRP1 was significantly lower in db/db mice than in db/m mice (*P*<0.01, Supplementary Figure 3A).

Moreover, we found that the expression of some kidney damage markers increased in db/db mice (Figure 2C, 2E and Supplementary Figure 3B). The expression of *Klf10* and *Klf11* showed the most significant increase in the proximal tubules and the PTS3 cluster, respectively. On the other hand, *Arg2* expression significantly increased in the LOH and CD clusters (Supplementary Figure 3B). The expression levels of *Txnip* and *Wfdc2* were significantly increased in the proximal tubules of db/db mice compared to those of db/m mice (*P*<0.01, Figure 2C). As Wfdc2 is a secretory protein associated with renal fibrosis, we chose it for further analysis in DKD patients. We compared *Wfdc2* expression in the morning urine of patients with DKD and NDKD controls. Western blotting showed that Wfdc2 expression was higher in the morning urine of patients with DKD than in that of controls (Figure 2D). Immunohistochemistry revealed high Wfdc2 expression in the lumen of the renal tubules and moderate Wfdc2 expression in the cells of the renal tubules, and the expression levels were higher in DKD patients than in NDKD controls (Figure 2D).

### Metabolic reprograming in db/db mice

**Figure 3:**
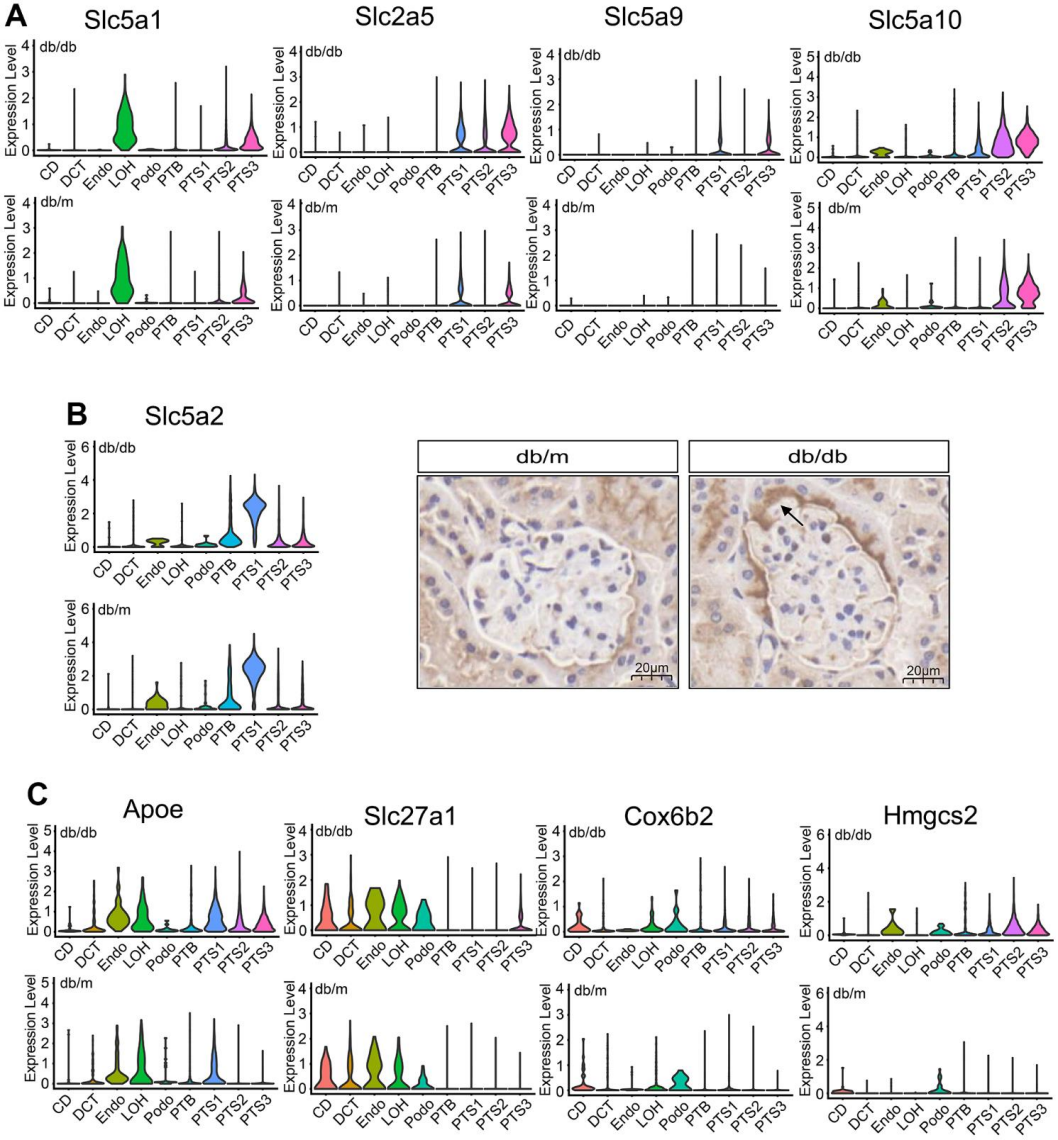
**Metabolic changes in the renal tubule cells of db/db and db/m mice.** (**A**) Violin plots showing the expression of glucose transporter in db/db and db/m mice. (**B**) Violin plots and immunohistochemical staining results of the expression of the Slc5a2 (SGLT2) transporter in db/db and db/m mice. Scale bar, 20 μm. (**C**) Violin plots showing the expression of Apoe, Slca27a1, Cox6b2, and Hmgcs2 transporters in db/db and db/m mice.

The expression levels of glucose reabsorption transporters (*Slc5a1, Slc5a2*, *Slc2a5, Slc5a9*, and *Slc5a10*) significantly increased in the proximal tubules of db/db mice compared with those in the proximal tubules of db/m mice (*P*<0.01) (Figure 3A, 3B). Reabsorption of glucose in the renal tubules is mainly completed through SGLT2 (*Slc5a2*) (90%) [12]. We performed immunohistochemical analysis to localize the expression of SGLT2 *(Slc5a2)* in the kidneys of db/db and db/m mice. SGLT2 was found to be expressed in the luminal membrane of proximal renal tubules and the urinary pole of Bowman’s capsule in db/db mice at a significantly higher level than in db/m mice (Several are the whole Bowman’s capsule) (Figure 3B).

Regarding lipid transport, *Apoe* expression level significantly increased in the proximal tubules and that of *Slc27a1* significantly increased in the PTS3 cluster of db/db mice than that of db/m mice (*P*<0.01) (Figure 3C). Additionally, the expression of *Cox6b2*, involved in the oxidative phosphorylation of glucose, significantly increased in db/db mice compared with that in db/m mice (*P*<0.01), especially in the proximal tubules, CD, and LOH (Figure 3C). *Hmgcs2*, which is involved in ketogenesis, was also highly expressed in the proximal tubules, endothelial cells, and podocytes of the kidneys of db/db mice compared with that in db/m mice (*P*<0.01, Figure 3C). Conversely, the expression levels of the amino acid transporters *Slc1a1, Slc3a1*, and *Slc13a1* were lower in the proximal tubules of db/db mice than in those of db/m mice (*P*<0.01, Supplementary Figure 3C). Transporters related to sugar and ketone metabolism were highly expressed in db/db mice; however, the expression of some key amino acid transporters decreased. These data suggest that db/db mice undergo metabolic remodeling, manifested as metabolic changes in glycolipid and amino acid levels.

### Single-cell chromatin accessibility analysis in db/db and db/m mice

**Figure 4:**
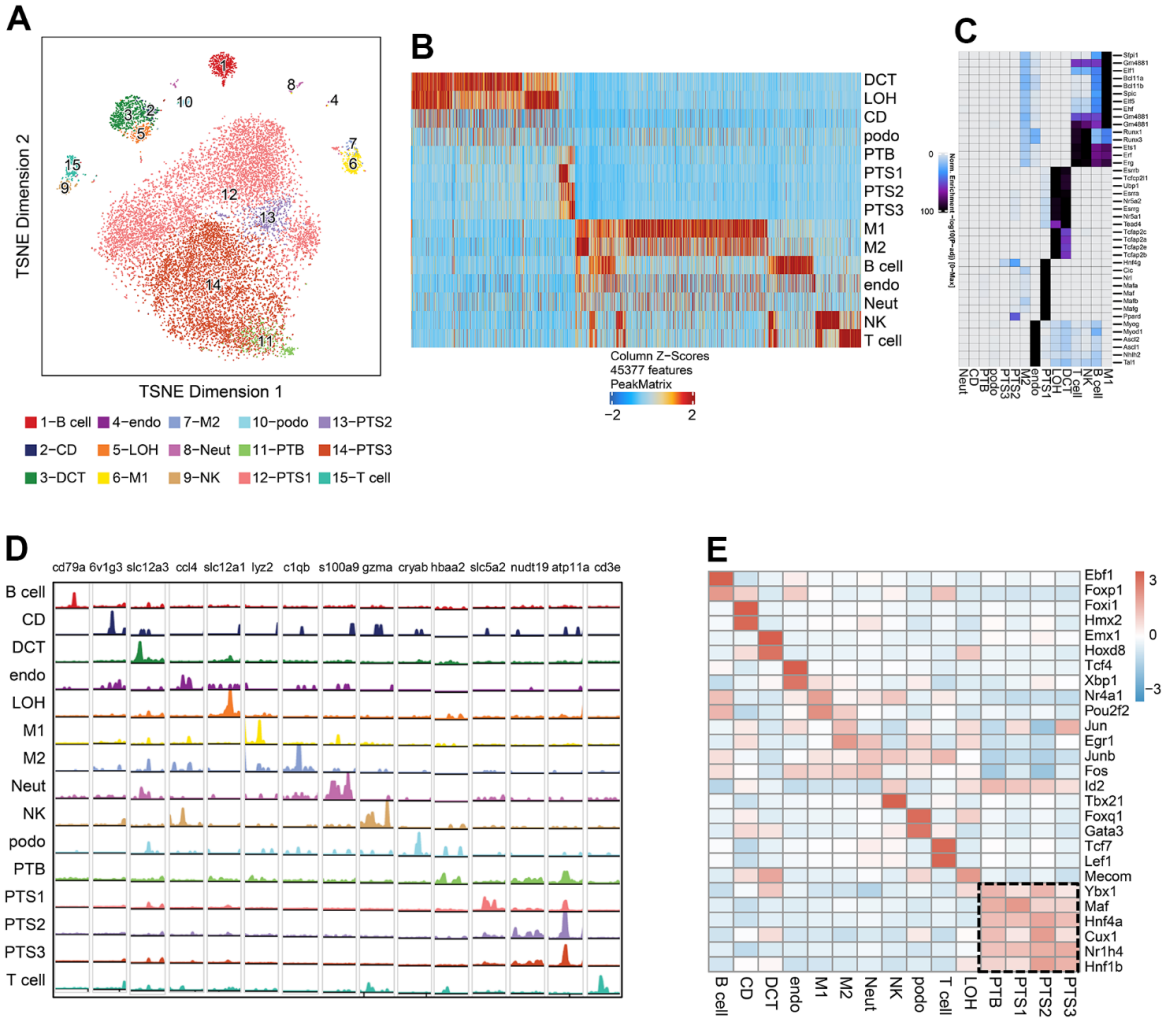
**Single-cell chromatin accessibility analysis of db/db and db/m mice.** (**A**) tSNE of scATAC-seq data from db/db and db/m mice kidney cells, colored by cell type. B cell, B lymphocytes; CD, collecting duct; DCT, distal convoluted tubule; Endo, endothelial cells; LOH, loop of Henle; M1, classical macrophage; M2, alternatively activated macrophage; NK, natural killer cell; Neut, neutrophil progenitor; Podo, podocyte; PTB, proximal tubule brush; PTS1, first segment of the proximal tubule; PTS2, second segment of the proximal tubule; PTS3, third segment of the proximal tubule; T cell, T lymphocytes. (**B**) Cell type-specific peaks in scATAC-seq data. (**C**) Cell type-specific transcription factor (TF) expression based on integrative analysis of scATAC-seq and scRNA-seq data. (**D**) Browser tracks of marker genes from scRNA-seq data in each cell type based on scATAC-seq data. (**E**) Heatmap showing the expression of cell type-specific positive TFs in each cluster, with the expression level indicated by color intensity.

We sequenced 16,660 cells from the kidneys of 3 db/db and 3 db/m mice, with an average of 7,069 fragments per cell; 14,968 cells were screened for further analysis after quality control. The cells from scATAC-seq were annotated as 15 clusters under the reference of defined cell types from the scRNA-seq data, which were visualized using tSNE (Figure 4A). We identified 85,212 differentially accessible open chromatin peaks (DAPs) across the 15 cell types (Figure 4B and Supplementary Table 5). The expression of 884 cell type specific TFs which are likely to bind to regions of high chromatin accessibility across the 15 clusters was observed (Figure 4C and Supplementary Table 6).

Browser tracks of marker genes from scRNA-seq data in each cell type were shown based on scATAC-seq data, including 6v*1g3* for CD, Slc*12a3* for DCT, *Ccl4* for Endo, *Slc12a1* for LOH, *Cryab* for Podo, and *Slc5a2* and *Atp11a* for the proximal tubules (Figure 4D). We identified the cell type specific positive TFs whose expression levels were positively correlated to changes in the accessibility of their corresponding motif by integrating the scRNA-seq and scATAC-seq data (Figure 4E and Supplementary Table 7). The expression of cell type specific positive TFs in each cluster is displayed as a heatmap (Figure 4E). Proximal tubule specific positive TFs included transcription factor Maf (*Maf*), hepatocyte nuclear factor 4-alpha (*Hnf4a*), homeobox protein cut-like 1 (*Cux1*), bile acid receptor (*Bar*/*Nr1h4*), and hepatocyte nuclear factor 1-beta (*Hnf1β*). The endothelial-specific positive TFs included transcription factor 4 (*Tcf4*) and X-box-binding protein 1 (*Xbp1*), and the podocyte-specific positive TFs included forkhead box protein Q1 (*Foxq1*) and GATA-3 (*Gata3*).

**Figure 5:**
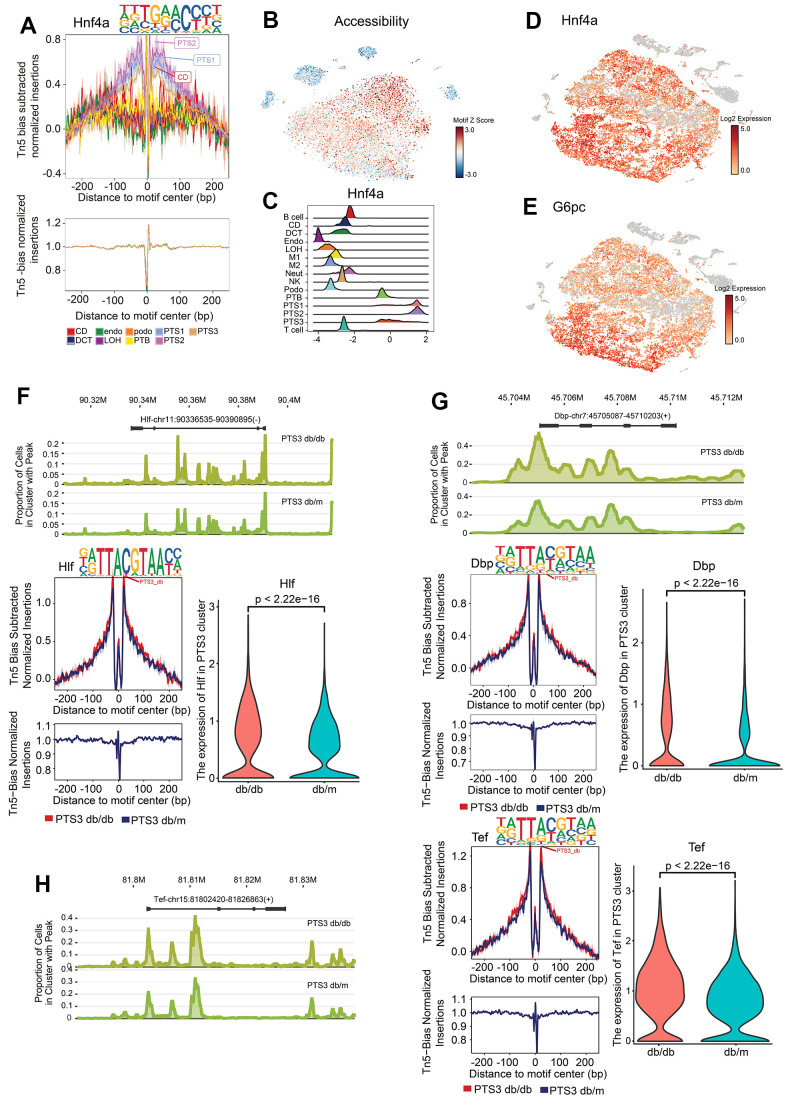
**Specific transcription factors in the proximal tubules.** (**A**) Footprinting of *Hnf4a*. (**B**) The chromatin accessibility of the region that *Hnf4a* bound was visualized using tSNE, with the accessibility level indicated by color intensity. (**C**) Differential motif enrichment analysis of *Hnf4a*: density map showing the distribution of motif deviation coefficient values. (**D**, **E**) Expression of *Hnf4a* and *G6pc* (target gene of *Hnf4a*), with the expression level indicated by color intensity. (**F**) Peak distribution, footprinting, and expression of *Hlf* in the PTS3 cluster of db/db and db/m mice. (**G**) Peak distribution, footprinting, and expression of *Dbp* in the PTS3 cluster of db/db and db/m mice. (**H**) Peak distribution, footprinting, and expression of *Tef* in the PTS3 cluster of db/db and db/m mice.

Footprinting showed the predicted precise binding location of *Hnf4a* and *Hnf1β*, and tSNE was used to visualize their expression in the proximal tubules (Figure 5A–5D and Supplementary Figure 4). *G6pc* is a target gene of *Hnf4a* [13], and its expression was consistent with that of *Hnf4a*, mainly being expressed in the proximal tubules (Figure 5E). *Rbm47* is a target gene of *Hnf1β* [14], and its expression was consistent with that of *Hnf1β* (Supplementary Figure 5E). Footprinting also showed the predicted precise binding location of *Maf, Cux1*, and *Nr1h4*, and the chromatin accessibility of the regions that *Maf*, *Cux1*, and *Nr1h4* bound were visualized using tSNE (Supplementary Figure 4F–4H).

### Circadian rhythm-associated TFs in db/db renal tubular cells

Hepatic leukemia factor (*Hlf*), D site-binding protein (*Dbp*), and thyrotroph embryonic factor (*Tef*) were found to bind to the regions with high chromatin accessibility in the PTS3 cluster of db/db mice more than that in db/m mice (*P*<0.01, Figure 5F–5H). *Hlf*, *Dbp*, and *Tef* belong to the PAR-domain basic leucine zipper (PAR bZip) family and serve as the output executors of the biological clock [15, 16].

**Figure 6:**
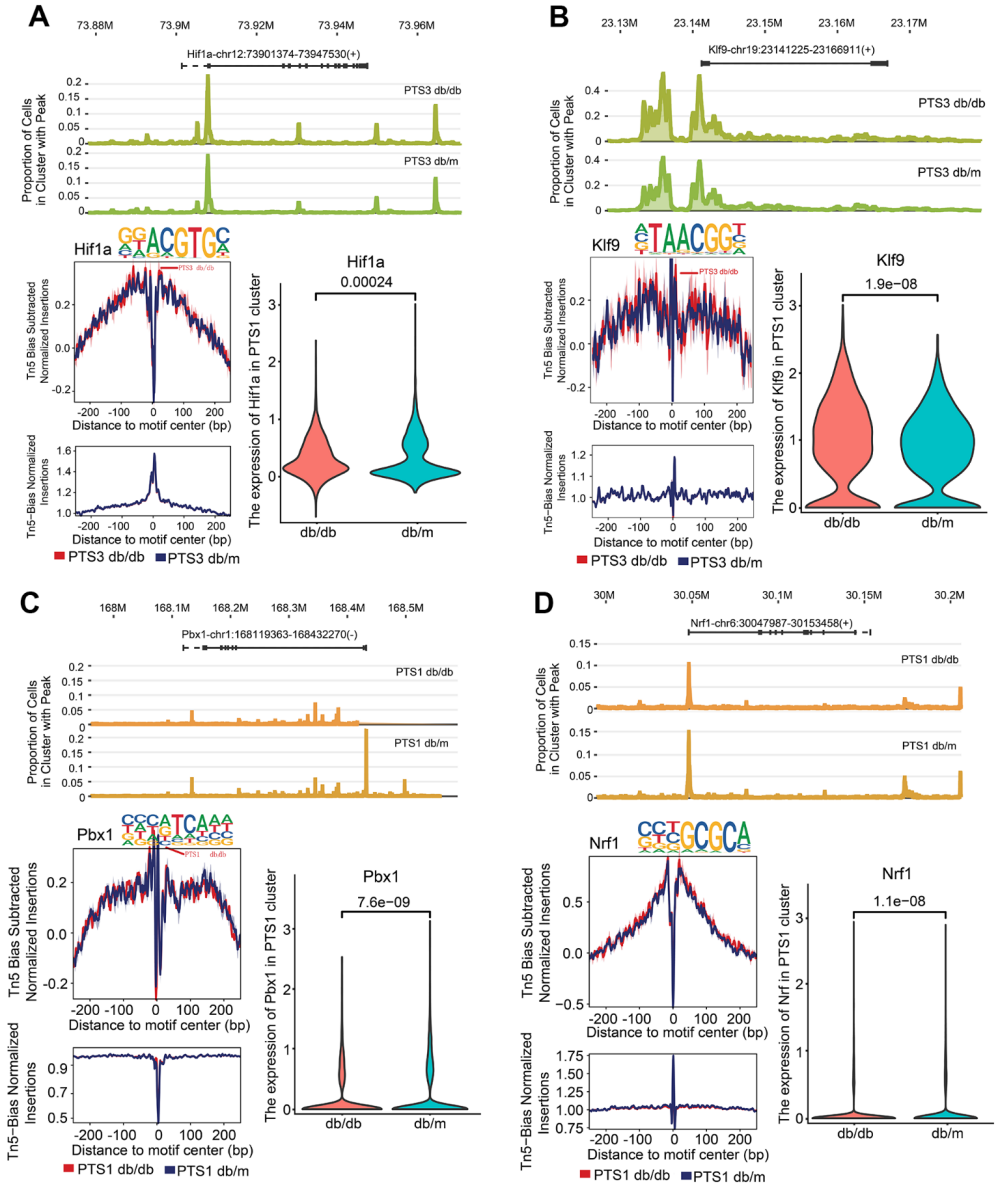
**Kidney injury-related transcription factor (TF) analysis in the PTS1 and PTS3 clusters.** (**A**) Peak distribution, footprinting, and expression of *Hif1*a in the PTS3 cluster of db/db and db/m mice. (**B**) Peak distribution, footprinting, and expression of *Klf9* in the PTS3 cluster of db/db and db/m mice. (**C**) Peak distribution, footprinting, and expression of *Pbx1* in the PTS1 cluster of db/db and db/m mice. (**D**) Peak distribution, footprinting, and expression of *Nrf1* in the PTS1 cluster of db/db and db/m mice.

Hypoxia-inducible factor 1-alpha (*Hif1α*) and Kruppel-like factor 9 (*Klf9*) showed higher chromatin accessibility of their promoter regions in the PTS3 cluster of db/db mice than in those of db/m mice (*P*<0.01, Figure 6A, 6B). Nuclear respiratory factor 1 (*Nrf1*) and pre-B-cell leukemia transcription factor 1 (*Pbx1*) showed lower chromatin accessibility of their promoter regions in the PTS1 cluster of db/db mice than in those of db/m mice (*P*<0.01). *Pbx1* is necessary for renal development and is involved in the activation of the nephrin promoter in the proximal tubules [17], while *Nrf1* acts as a cytoprotective factor in cells and plays a regulatory role in diabetes by modulating the level of glutathione [18] (Figure 6C, 6D).

## DISCUSSION

The pathogenesis of DKD is complex, and the role of the renal tubules is gaining increasing attention. We performed scATAC-seq and scRNA-seq analyses in db/db and db/m mice, focusing on differential gene expression and cell type-specific TFs in the renal tubules. Our data showed that in a high glucose environment, the renal tubules undergo an adaptive response. This is first manifested by increased glucose reabsorption, with significant upregulation of *SGLT2* in the proximal tubules, particularly with Bowman’s capsule reflux of *SGLT2* expression. We observed significantly increased expression of *SGLT2* in the urine pole of Bowman’s capsule in db/db mice, which may be related to tubular metaplasia of the Bowman’s capsule under high glucose conditions [19]. Additionally, the expression of other glucose transporters (*Slc5a1*, *Slc2a5*, *Slc5a9*, and *Slc5a10*) also significantly increased (*P*<0.01), and some TFs that promote glucose transport were significantly upregulated in the proximal tubules. Under hyperglycemic conditions, renal tubular reabsorption of glucose and sodium as well as passive reabsorption of chloride and water increase. The glomeruli undergo hyperfiltration in response to increased reabsorption by the renal tubules, which can manifest as an increase in the GFR [7, 8].

The increase in tubular reabsorption of glucose leads to a large influx of glucose into the cells, inducing changes in glucose metabolism pathways and resulting in the accumulation of glucose sub-metabolites within the cells [20]. Furthermore, we observed a significant increase in the expression of *Apoe* and *Slc27a1* transporters involved in the reabsorption of lipids in the renal tubules (*P*<0.01), This may further promote lipid accumulation and result in cell toxicity. While the expression of glucose and lipid transporters increased, some critical amino acid transporters in the proximal renal tubules (*Slc1a1*, *Slc3a1*, *Slc13a1*) decreased (*P*<0.01), which may further exacerbate the metabolic imbalance in the proximal tubules during DKD.

Under DKD conditions, signaling pathways such as PPAR, TGF-β, MAPK, and NLRP3 are activated in the renal tubules, and they transition from adaptive to pathological changes. We observed that *klf10* and *klf11*, which encode factors involved in the TGF-β signaling pathway [21] and inhibit cell growth, regulating extracellular matrix, and inducing cell apoptosis [22, 23] are significantly upregulated in the proximal tubules. Consistently, the cell numbers in the PTS3 and PTS2 clusters were significantly lower in db/db mice than in db/m mice, this could be attributed to increased *Klf10* and *Klf11* expression. Additionally, the expression of *Txnip,* which can promote inflammatory in high glucose environment [24] and *Arg2*, which can induce the production of reactive oxygen species [25], were also significantly higher in db/db mice. As renal tubular pathological changes progress, expression of the renal tubular fibrosis protein *Wfdc2* was significantly increased in both db/db mice and the urine and kidneys of patients with DKD. Wfdc2 may function as a protease inhibitor, which can inhibit serine protease activity and slow down the degradation of type I collagen, thereby promoting renal fibrosis [26]. These results indicate there are significant pathological changes in the kidney in DKD.

In a high glucose environment, the enhanced reabsorption function of renal tubules also leads to increased oxygen consumption by tubular cells, resulting in renal hypoxia [27]. In line with cellular adaptation to a hypoxic state, we found that some specific regions with high chromatin accessibility in the proximal tubules of db/db mice may bind to *HIF-1α*, additionally, sc-RNA seq showed that *HIF-1α* expression increased in the proximal tubules of db/db mice.

*HIF-1α* can heterodimerize with *BMAL* to upregulate the expression of *DBP* [28–30]. Similarly, some specific regions with high chromatin accessibility in the proximal tubules of db/db mice were suggested to bind to *Hlf*, *Dbp*, *Tef* and *klf9*, and sc-RNA seq showed that *Hlf*, *Dbp*, *Tef* and *klf9* expression increased in the proximal tubules of db/db mice.

*Dbp*, *Tef*, and *Hlf* can accelerate cell apoptosis caused by Bcl-2-interacting killer (*Bik*) through oxidative stress [31]. Moreover, *Dbp* overexpression can inhibit the proliferation of mesangial cells and prolong the cell cycle in rat fibroblasts [32]. The increased chromatin accessibility and *Dbp* expression may also be attributed to the decrease in PTS3 cluster cell numbers.

*Hlf*, *Dbp*, and *Tef* are transmitters of circadian rhythm signals *in vivo* [16, 33] and *Klf9* is regulated by circadian rhythm. Over 1,000 renal transcripts, including podocin (*Nphs2), Aqp2, Aqp4, Aqp8*, and solute carrier family 2, facilitated glucose transporter member 9 (*Slc2a9*, *Glut9*), have been found to follow circadian rhythm patterns [34]. The biological clock regulates renal functions, including GFR, renal plasma flow, renal resorption of water, absorption and excretion of major urinary solutes, renal glucose homeostasis, profibrotic mechanisms, and hypoxic signaling [35–37]. Indeed, some research indicates that circadian rhythm has a significant effect on GFR. In podocytes of mice lacking *BMAL1*, the GFR circadian rhythm is lost and changes to a 12-hour periodic hypercircadian rhythm [38]. *Klf9* dysregulation due to altered circadian rhythm can contribute to podocyte damage in high-glucose conditions [39, 40]. This suggests that the intrinsic “biological clock” of the kidney is essential to kidney function and disruption of circadian rhythm could have detrimental effects.

In summary, this preliminary study revealed differential gene expression and TFs in db/db mice that may elucidate the mechanisms underlying the pathological changes in renal tubular cells in DKD at the single-cell level.

We show that the hyperglycemic environment of DKD triggers various stress responses, enhances crosstalk between podocytes and tubular cells in the proximal tubules, and activates inflammation, hypoxia, and fibrosis signaling pathways. Of particular interest, the expression of the fibrosis marker Wfdc2 was increased in both db/db mice and in the morning urine and kidneys of DKD patients, giving translational validity to our results. These findings suggests that pathological changes in the renal tubules may play a decisive role in the onset and progression of DKD, future studies with larger sample sizes are needed.

## MATERIALS AND METHODS

### Model animals

All animal experiments were performed in accordance with the experimental guidelines of the Experimental Animal Ethics Committee of Zhengzhou University (approval number ZZU-LAC20201013). Three 16-week-old male db/db mice and three 16-week-old male db/m mice were purchased from the Jiangsu Jicui Yaokang Biotechnology Company. The baseline information of db/db and db/m mice is listed in Supplementary Table 8.

### Specimens from DKD patients and NDKD controls

The DKD urine and kidney specimens were obtained from hospitalized patients who were diagnosed with Diabetic Nephropathy based on the pathology of renal biopsies in the First Affiliated Hospital of Zhengzhou University (n=3). The NDKD kidney specimens were collected from patients undergoing surgical treatment for renal carcinoma (n=3). Specimens used in this study were collected 5cm from the tumor. This study was approved by the Ethics Committee of the First Affiliated Hospital of Zhengzhou University.

### Preparation of single-cell suspensions

Mouse kidney tissues were placed in a sterile Petri dish with 10 mL precooled (4° C) 1× Dulbecco’s phosphate-buffered saline (PBS, Thermo Fisher Scientific, Waltham, MA, USA). After washing twice, the tissues were cut to obtain 1- to 2-mm^3^ pieces, placed in fresh dishes with lysis buffer that contains 0.25% trypsin (Thermo Fisher Scientific) and 10 μg/mL DNase I (Sigma-Aldrich, St. Louis, MO, USA), and lysed in a 50-mL centrifuge tube in a water bath at 37° C. An equal volume of the complete medium (Thermo Fisher Scientific) was added to stop the lysis. The buffer was passed through a pre-wetted 70-μm filter (Millipore, Burlington, MA, USA), and the filtrate was collected in a fresh 50-mL centrifuge tube. The filter was rinsed with 10 mL complete medium, whereas the filtrate was collected in the same centrifuge tube and centrifuged at 100 ×*g* for 5 min at 25° C. The supernatant was discarded, and 5 mL 1× Dulbecco’s PBS containing 2% fetal bovine serum (FBS, Gibco, Grand Island, NY, USA) was added to resuspend the cells. The suspension was centrifuged at 100 ×*g* for 5 min, the supernatant was discarded, and 3 mL precooled (4° C) 1× erythrocyte lysis buffer (Thermo Fisher Scientific) was added, followed by gentle blowing and mixing. The suspension was incubated at 25° C for 3 min and centrifuged at 300 ×*g* for 5 min, and the supernatant was discarded. Next, 5 mL precooled 1× Dulbecco’s PBS containing 2% FBS was added, followed by gentle blowing and mixing; this mixture was centrifuged at 300 ×*g* for 5 min. These steps were repeated until a single-cell suspension was obtained. The suspension was placed on ice for cell counting.

### scRNA-seq

scRNA-seq was performed using a 10× Genomics platform. The single-cell suspension, 10× barcode gel magnetic beads, and oil were added to the different chambers of chromium chip A (Chromium™ Chip A Single Cell Kit, 120236, 10× Genomics, Pleasanton, CA, USA) to generate single-cell gel beads-in-emulsion (GEM) through the microfluidic “double cross” system. The magnetic beads on the gel contained 30-nt oligo primers to reverse-transcribe poly-A RNA into cDNA chains with barcode and unique molecular identifier information. The purified cDNA chains were amplified via PCR and quantified using Qubit. Fragment size was detected using an Agilent 2100 system (Agilent Technologies, Santa Clara, CA, USA).

Optimal fragments obtained by enzyme digestion were screened using magnetic beads (SPRIselect Reagent Kit, B23318, Beckman Coulter, Pasadena, CA, USA). A cDNA library containing the P5 and P7 linkers was constructed using PCR after terminal repair and the addition of A and adapter-ligated Read2 sequencing primers (Chromium i7 Multiplex Kit, 120262, 10× Genomics; Chromium™ Single Cell 5′ GEM, Library and Gel Bead Kit v3, 1000006, 10× Genomics).

The library was purified using magnetic beads, the concentration of the fragments in the library was detected using Qubit, and the fragment size was measured using Agilent 2100. T and B cell receptor libraries were constructed similarly (Chromium™ Single Cell V(D)J Enrichment kit, 1000005 and 000016, 10× Genomics). The library was sequenced and analyzed using the Illumina sequencing platform (Illumina, San Diego, CA, USA). Sequencing was controlled using the data collection software provided by Illumina, and real-time data analysis was performed.

### scATAC-seq

scATAC-seq was performed using the 10× Chromium™ Single Cell ATAC platform. The cell suspension was added to a 1.5-mL centrifuge tube, followed by centrifugation at 300 ×*g* (4° C) for 5 min. The supernatant was discarded, and lysis buffer was added to the pellet, followed by gentle blowing to resuspend the nuclei and incubation on ice for 15 min. The proportion of live cells was monitored using a fluorescent cell counter until it dropped to 0%–5%. Finally, the nuclei were washed and counted using a cell counter.

Nuclei were harvested and immediately analyzed on the 10× Chromium™ Single Cell ATAC platform. The nuclear suspension, 10× barcode gel magnetic beads, and oil were added to the different chambers of chromium chip H (Chromium™ Chip H Single Cell Kit, 1000161, 10× Genomics) to generate single-cell GEM through the microfluidic system. The transposase with the DNA adapter came into contact with the DNA where chromatin was loosely wound, resulting in a transposition reaction at these sites. As a result, a DNA fragment was cut from the chromatin and formed a free DNA fragment, which was then bound by the DNA adapter. The Read 1N sequence attached to the DNA label strand on the gel bead was complementary to the linker sequence on the DNA fragment and was annealed and adhered. The GEM was then transferred into the thermal cycler. Under the action of polymerase, the DNA fragments were extended to 10x the Barcode sequences, connected to the P5 sequences, used for library construction (Chromium™ Single Cell ATAC GEM, Library and Gel Bead Kit v1.1), and sequenced.

Qubit was used to measure the concentration, and the Agilent 4200 system was used to detect the fragment size. The library was sequenced and analyzed using the Illumina sequencing platform. Sequencing was controlled using the data collection software provided by Illumina, and real-time data analysis was conducted.

### Data processing and quality control

Cellranger (cell range: 3.0.1) was used to map Raw FASTQ files to the mm10 reference genome (Ensembl GRCm38,93), and the scRNA-seq data were converted from the fastq type to a cell expression matrix, retaining cells with >200 expressed genes. The effects on the cell cycle were negligible and could not be detected in this study.

### Dimension reduction, batch effect correction, and clustering

The top 2000 genes with the largest variation were identified based on the average value and dispersion (variance/average) of all genes in each sample. We used the combination of canonical correlation analysis and mutual nearest neighbors to correct for batch effects. Principal component analysis was performed for dimension reduction, and the first 20 dimensions were used for subsequent analysis. Normalized data were clustered and analyzed using the Louvain algorithm (resolution: 0.5) and visualized using tSNE.

### Cell-type annotation and marker genes

The cell type was annotated manually using singleR and marker gene software referring to the marker gene list (Supplementary Table 2). The marker genes of all clusters were analyzed using the Wilcoxon algorithm (P < 0.05). The genes in each cluster that showed high specific expression (log-fold change [FC] > 0.25) and were expressed in at least 20% of cells were selected as significant marker genes of the corresponding cluster, displayed using R pheatmap, and visualized using tSNE.

### DEG and functional enrichment analyses

The DEGs between different clusters and groups were analyzed using the FindMarkers function in Seurat, followed by KEGG functional enrichment analysis. Gene expression profiles were visualized using ggplot, and heatmaps were constructed using the R pheatmap package.

### Quality control, doublet filtration, and dimension reduction

We used CellRanger to convert the scATAC-seq data from the six mouse samples from the FASTQ format to the fragment format. We calculated the transcription start site (TSS) enrichment score of each cell (average accessibility in the 50-bp region centered on each single-base TSS position divided by the average accessibility [±1900–2000 bp] of the TSS flank position). Cells with a TSS enrichment score of >4 and >3000 fragments size were eliminated (Supplementary Figure 5). ArchR was used to ascertain whether the cell is a “double” (i.e., two different genotypes within a cell), and double scores were allotted to each cell accordingly. Double cells were filtered at a cut-off rate of 5%. ArchR was used to reduce the hierarchical dimensionality, and harmony was used to correct the sample batch effects. tSNE was used for data visualization.

### Clustering and annotation

Gene scores were evaluated based on the open chromatin data obtained after scATAC-seq and calculated using the distance-weighted openness model. The gene score represents the extent to which gene expression is predicted by the regulatory elements near the gene. First, the data were integrated by mapping the gene score matrices of the scATAC-seq data to those of the scRNA-seq data [41]. Second, the Seurat clustering method was used to annotate the cell types in the scATAC-seq data based on the defined clusters of the scRNA-seq data. ArchR was used to identify marker genes according to the gene scores. Significant marker genes of each cluster were identified (false-discovery rate ≤ 0.01 and log2 FC ≥ 1.25). The gene scores of each cell were embedded into tSNE to visualize the marker genes from the scATAC-seq data.

### Peak calling, marker peak identification, and motif enrichment

ArchR was used for peak calling of the scATAC data, which mainly involved the construction of pseudo bulk replicates (i.e., data that imitate bulk ATAC-seq after all single cells were merged) from multiple pseudo bulk samples in each cluster. Marker peak analysis of each cluster was performed similar to cluster marker gene identification. After obtaining the marker peaks specifically enriched in each cluster [42], the getMarkerFeatures function in ArchR was used for motif enrichment analysis to predict the TFs associated with transcription-binding events and open chromatin sites [43]. The browser tracks of these TFs were drawn.

### Footprint analysis, distribution of the chromVAR deviation score, and positive TF regulators

TF footprints can predict the precise binding position of TFs at specific locations. We used ArchR to analyze the Tn5 deviation footprints. Due to the deviation of Tn5 transposase insertion sequences, the distribution of the chromVAR deviation scores of TFs was assessed using chromVAR. Normalization was performed to remove the bias associated with Tn5 from the coverage signal. ArchR was used to identify the positive TF regulators in the scATAC data. We correlated the chromVAR deviation Z scores of the TF motifs with the expression of TF genes from low-overlap aggregates and selected the TFs whose motif accessibility was positively correlated with TF expression. Target genes specific to the TFs of interest were predicted using the TRRUST v2 and Cistrome Data Browser online database [44, 45].

### Immunohistochemical analysis

First, the paraffin sections are dewaxed and hydrated by heating at 60° C for 30 min, treatment with xylene for 15 min, and treatment with a gradual ethanol series of 100%, 95%, 85% and 75% ethanol for 5 min each. The sections then underwent antigen retrieval using the microwave oven repair method. Sections were incubated with 3% H_2_O_2_ to inactivate endogenous peroxidases and then blocked with 2% goat serum for 30 min. Subsequently, the sections were incubated overnight at 4° C with primary antibodies (SGLT2 antibody, BiCell Scientific, BC-20802; Wfdc antibody, Sigma-Aldrich, HPA042302). Color rendering was performed using diaminobenzidine (DAB), and the sections were photographed.

### Western blotting

The morning urine of DKD patients and healthy controls were collected and filtered using a 0.2-μm filter and then concentrated 10-fold using the Centrifugal Filter Unit (Merck Millipore, UFC9010). The samples were run on a 15% SDS-PAGE gel at 120 V for 40 min. The gel was transferred to a methanol-activated PVDF membrane and run at 250 mA for 30 min and then blocked with 5% skimmed milk. The PVDF membrane was then incubated at 4° C overnight with the primary antibody (WFDC2, Servicebio, Wuhan, China, GB111377). Subsequently, a secondary antibody was applied, followed by incubation, washing, and imaging.

### Transmission electron microscopy (TEM)

The kidney specimens were fixed in glutaraldeyde immediately after obtained from db/db and db/m mice. Ultrathin sections were cut with an ultramicrotome, stained with 2% (wt/vol) uranyl acetate and lead citrate, and examined with a JEOL JEM-1400 Plus transmission electron microscope.

### Statistical analysis

All statistical analyses were performed using R software and corrected using the false-discovery rate, *P*-value < 0.05 was considered statistically significant.

### Data availability statement

Single cell sequencing data in FASTQ format are available at the Genome Sequence Archive (GSA) for Human at the BIG Data Center, Beijing Institute of Genomics (accession number PRJCA014746).

## Supplementary Material

Supplementary Figures

Supplementary Table 1

Supplementary Table 2

Supplementary Table 3

Supplementary Table 4

Supplementary Table 5

Supplementary Table 6

Supplementary Table 7

Supplementary Table 8

## References

[1] Magliano DJ, Boyko EJ. IDF Diabetes Atlas 10th edition scientific committee. IDF DIABETES ATLAS. 10th ed. Brussels: International Diabetes Federation. 2021. 35914061

[2] Selby NM, Taal MW. An updated overview of diabetic nephropathy: Diagnosis, prognosis, treatment goals and latest guidelines. Diabetes Obes Metab. 2020 (Suppl 1); 22:3–15. 10.1111/dom.14007 32267079

[3] MacIsaac RJ, Ekinci EI. Progression of Diabetic Kidney Disease in the Absence of Albuminuria. Diabetes Care. 2019; 42:1842–4. 10.2337/dci19-0030 31540958

[4] Ix JH, Shlipak MG. The Promise of Tubule Biomarkers in Kidney Disease: A Review. Am J Kidney Dis. 2021; 78:719–27. 10.1053/j.ajkd.2021.03.026 34051308 PMC8545710

[5] Brocco E, Fioretto P, Mauer M, Saller A, Carraro A, Frigato F, Chiesura-Corona M, Bianchi L, Baggio B, Maioli M, Abaterusso C, Velussi M, Sambataro M, et al. Renal structure and function in non-insulin dependent diabetic patients with microalbuminuria. Kidney Int Suppl. 1997; 63:S40–4. 9407419

[6] Dalla Vestra M, Saller A, Bortoloso E, Mauer M, Fioretto P. Structural involvement in type 1 and type 2 diabetic nephropathy. Diabetes Metab. 2000 (Suppl 4); 26:8–14. 10922968

[7] Vallon V, Thomson SC. The tubular hypothesis of nephron filtration and diabetic kidney disease. Nat Rev Nephrol. 2020; 16:317–36. 10.1038/s41581-020-0256-y 32152499 PMC7242158

[8] Zeni L, Norden AG, Cancarini G, Unwin RJ. A more tubulocentric view of diabetic kidney disease. J Nephrol. 2017; 30:701–17. 10.1007/s40620-017-0423-9 28840540 PMC5698396

[9] Vallon V, Blantz RC, Thomson S. Glomerular hyperfiltration and the salt paradox in early [corrected] type 1 diabetes mellitus: a tubulo-centric view. J Am Soc Nephrol. 2003; 14:530–7. 10.1097/01.asn.0000051700.07403.27 12538755

[10] Thomson SC, Vallon V, Blantz RC. Kidney function in early diabetes: the tubular hypothesis of glomerular filtration. Am J Physiol Renal Physiol. 2004; 286:F8–15. 10.1152/ajprenal.00208.2003 14656757

[11] Vallon V, Thomson SC. Renal function in diabetic disease models: the tubular system in the pathophysiology of the diabetic kidney. Annu Rev Physiol. 2012; 74:351–75. 10.1146/annurev-physiol-020911-153333 22335797 PMC3807782

[12] Boillot D, Assan R, Dardenne M, Debray-Sachs M, Bach JF. T-lymphopenia and T-cell imbalance in diabetic db/db mice. Diabetes. 1986; 35:198–203. 10.2337/diab.35.2.198 3510925

[13] Kimura M, Tanaka S, Isoda F, Sekigawa K, Yamakawa T, Sekihara H. T lymphopenia in obese diabetic (db/db) mice is non-selective and thymus independent. Life Sci. 1998; 62:1243–50. 10.1016/s0024-3205(98)00054-x 9570339

[14] Gyimesi G, Pujol-Giménez J, Kanai Y, Hediger MA. Sodium-coupled glucose transport, the SLC5 family, and therapeutically relevant inhibitors: from molecular discovery to clinical application. Pflugers Arch. 2020; 472:1177–206. 10.1007/s00424-020-02433-x 32767111 PMC7462921

[15] Hirota K, Sakamaki J, Ishida J, Shimamoto Y, Nishihara S, Kodama N, Ohta K, Yamamoto M, Tanimoto K, Fukamizu A. A combination of HNF-4 and Foxo1 is required for reciprocal transcriptional regulation of glucokinase and glucose-6-phosphatase genes in response to fasting and feeding. J Biol Chem. 2008; 283:32432–41. 10.1074/jbc.M806179200 18805788

[16] Zhao J, Lupino K, Wilkins BJ, Qiu C, Liu J, Omura Y, Allred AL, McDonald C, Susztak K, Barish GD, Pei L. Genomic integration of ERRγ-HNF1β regulates renal bioenergetics and prevents chronic kidney disease. Proc Natl Acad Sci USA. 2018; 115:E4910–9. 10.1073/pnas.1804965115 29735694 PMC6003475

[17] Savino M, Guida CC, Nardella M, Murgo E, Augello B, Merla G, De Cosmo S, Savino AF, Tarquini R, Cei F, Aucella F, Mazzoccoli G. Circadian Genes Expression Patterns in Disorders Due to Enzyme Deficiencies in the Heme Biosynthetic Pathway. Biomedicines. 2022; 10:3198. 10.3390/biomedicines10123198 36551954 PMC9775071

[18] Schurhoff N, Toborek M. Circadian rhythms in the blood-brain barrier: impact on neurological disorders and stress responses. Mol Brain. 2023; 16:5. 10.1186/s13041-023-00997-0 36635730 PMC9835375

[19] Pan L, Gross KW. Transcriptional regulation of renin: an update. Hypertension. 2005; 45:3–8. 10.1161/01.HYP.0000149717.55920.45 15545507

[20] Legouis D, Faivre A, Cippà PE, de Seigneux S. Renal gluconeogenesis: an underestimated role of the kidney in systemic glucose metabolism. Nephrol Dial Transplant. 2022; 37:1417–25. 10.1093/ndt/gfaa302 33247734

[21] Wang Z, Fu Y, do Carmo JM, da Silva AA, Li X, Mouton A, Omoto AC, Sears J, Hall JE. Transient receptor potential cation channel 6 contributes to kidney injury induced by diabetes and hypertension. Am J Physiol Renal Physiol. 2022; 322:F76–88. 10.1152/ajprenal.00296.2021 34866402 PMC8742740

[22] Haythorne E, Lloyd M, Walsby-Tickle J, Tarasov AI, Sandbrink J, Portillo I, Exposito RT, Sachse G, Cyranka M, Rohm M, Rorsman P, McCullagh J, Ashcroft FM. Altered glycolysis triggers impaired mitochondrial metabolism and mTORC1 activation in diabetic β-cells. Nat Commun. 2022; 13:6754. 10.1038/s41467-022-34095-x 36376280 PMC9663558

[23] Spittau B, Krieglstein K. Klf10 and Klf11 as mediators of TGF-beta superfamily signaling. Cell Tissue Res. 2012; 347:65–72. 10.1007/s00441-011-1186-6 21574058

[24] Papadakis KA, Krempski J, Svingen P, Xiong Y, Sarmento OF, Lomberk GA, Urrutia RA, Faubion WA. Krüppel-like factor KLF10 deficiency predisposes to colitis through colonic macrophage dysregulation. Am J Physiol Gastrointest Liver Physiol. 2015; 309:G900–9. 10.1152/ajpgi.00309.2015 26472224 PMC4669350

[25] Zakeri S, Aminian H, Sadeghi S, Esmaeilzadeh-Gharehdaghi E, Razmara E. Krüppel-like factors in bone biology. Cell Signal. 2022; 93:110308. 10.1016/j.cellsig.2022.110308 35301064

[26] LeBleu VS, Teng Y, O’Connell JT, Charytan D, Müller GA, Müller CA, Sugimoto H, Kalluri R. Identification of human epididymis protein-4 as a fibroblast-derived mediator of fibrosis. Nat Med. 2013; 19:227–31. 10.1038/nm.2989 23353556 PMC4457508

[27] He Q, Li Y, Zhang W, Chen J, Deng W, Liu Q, Liu Y, Liu D. Role and mechanism of TXNIP in ageing-related renal fibrosis. Mech Ageing Dev. 2021; 196:111475. 10.1016/j.mad.2021.111475 33781783

[28] Morris SM Jr, Gao T, Cooper TK, Kepka-Lenhart D, Awad AS. Arginase-2 mediates diabetic renal injury. Diabetes. 2011; 60:3015–22. 10.2337/db11-0901 21926276 PMC3198072

[29] Iacobini C, Vitale M, Haxhi J, Pesce C, Pugliese G, Menini S. Mutual Regulation between Redox and Hypoxia-Inducible Factors in Cardiovascular and Renal Complications of Diabetes. Antioxidants (Basel). 2022; 11:2183. 10.3390/antiox11112183 36358555 PMC9686572

[30] Hogenesch JB, Gu YZ, Jain S, Bradfield CA. The basic-helix-loop-helix-PAS orphan MOP3 forms transcriptionally active complexes with circadian and hypoxia factors. Proc Natl Acad Sci USA. 1998; 95:5474–9. 10.1073/pnas.95.10.5474 9576906 PMC20401

[31] Matsui MS, Pelle E, Dong K, Pernodet N. Biological Rhythms in the Skin. Int J Mol Sci. 2016; 17:801. 10.3390/ijms17060801 27231897 PMC4926335

[32] He X, Zeng X. LncRNA SNHG16 Aggravates High Glucose-Induced Podocytes Injury in Diabetic Nephropathy Through Targeting miR-106a and Thereby Up-Regulating KLF9. Diabetes Metab Syndr Obes. 2020; 13:3551–60. 10.2147/DMSO.S271290 33116706 PMC7549883

[33] Peek CB, Levine DC, Cedernaes J, Taguchi A, Kobayashi Y, Tsai SJ, Bonar NA, McNulty MR, Ramsey KM, Bass J. Circadian Clock Interaction with HIF1α Mediates Oxygenic Metabolism and Anaerobic Glycolysis in Skeletal Muscle. Cell Metab. 2017; 25:86–92. 10.1016/j.cmet.2016.09.010 27773696 PMC5226863

[34] Firsov D, Bonny O. Circadian rhythms and the kidney. Nat Rev Nephrol. 2018; 14:626–35. 10.1038/s41581-018-0048-9 30143787

[35] Zlacká J, Zeman M. Glycolysis under Circadian Control. Int J Mol Sci. 2021; 22:13666. 10.3390/ijms222413666 34948470 PMC8703893

[36] Wang Y, Guo H, He F. Circadian disruption: from mouse models to molecular mechanisms and cancer therapeutic targets. Cancer Metastasis Rev. 2023; 42:297–322. 10.1007/s10555-022-10072-0 36513953

[37] Solocinski K, Gumz ML. The Circadian Clock in the Regulation of Renal Rhythms. J Biol Rhythms. 2015; 30:470–86. 10.1177/0748730415610879 26527094 PMC4892112

[38] Ansermet C, Centeno G, Nikolaeva S, Maillard MP, Pradervand S, Firsov D. The intrinsic circadian clock in podocytes controls glomerular filtration rate. Sci Rep. 2019; 9:16089. 10.1038/s41598-019-52682-9 31695128 PMC6838779

[39] Johnston JG, Pollock DM. Circadian regulation of renal function. Free Radic Biol Med. 2018; 119:93–107. 10.1016/j.freeradbiomed.2018.01.018 29360554 PMC6052790

[40] Castelo-Szekely V, Arpat AB, Janich P, Gatfield D. Translational contributions to tissue specificity in rhythmic and constitutive gene expression. Genome Biol. 2017; 18:116. 10.1186/s13059-017-1222-2 28622766 PMC5473967

[41] Jiang H, Li J, He X, Xue J, Liang S, Liu S, Gao F, Qu N, Liu H, Chen L. D-site binding protein regulates cell proliferation through mediating cell cycle progression in rat mesangial cells. Tissue Cell. 2019; 61:35–43. 10.1016/j.tice.2019.08.006 31759405

[42] Lun A, Andrews JM, Dundar F, Bunis D. Using SingleR to annotate single-cell RNA-seq data. 2020; 19363:713.

[43] Stuart T, Butler A, Hoffman P, Hafemeister C, Papalexi E, Mauck WM 3rd, Hao Y, Stoeckius M, Smibert P, Satija R. Comprehensive Integration of Single-Cell Data. Cell. 2019; 177:1888–902.e21. 10.1016/j.cell.2019.05.031 31178118 PMC6687398

[44] Zhang Y, Liu T, Meyer CA, Eeckhoute J, Johnson DS, Bernstein BE, Nusbaum C, Myers RM, Brown M, Li W, Liu XS. Model-based analysis of ChIP-Seq (MACS). Genome Biol. 2008; 9:R137. 10.1186/gb-2008-9-9-r137 18798982 PMC2592715

[45] Schep AN, Wu B, Buenrostro JD, Greenleaf WJ. chromVAR: inferring transcription-factor-associated accessibility from single-cell epigenomic data. Nat Methods. 2017; 14:975–8. 10.1038/nmeth.4401 28825706 PMC5623146

