# Supplementary material for: Analysis of potential biomarkers for diabetic kidney disease based on single-cell RNA-sequencing integrated with a single-cell sequencing assay for transposase-accessible chromatin
Source: Aging (Albany NY). 2023 Oct 11;15(19):10681–704. doi: 10.18632/aging.205107 (PMC10599739; doi:10.18632/aging.205107)
Supplement: Supplementary Figures [file aging-15-205107-s001.pdf]

SUPPLEMENTARY FIGURES

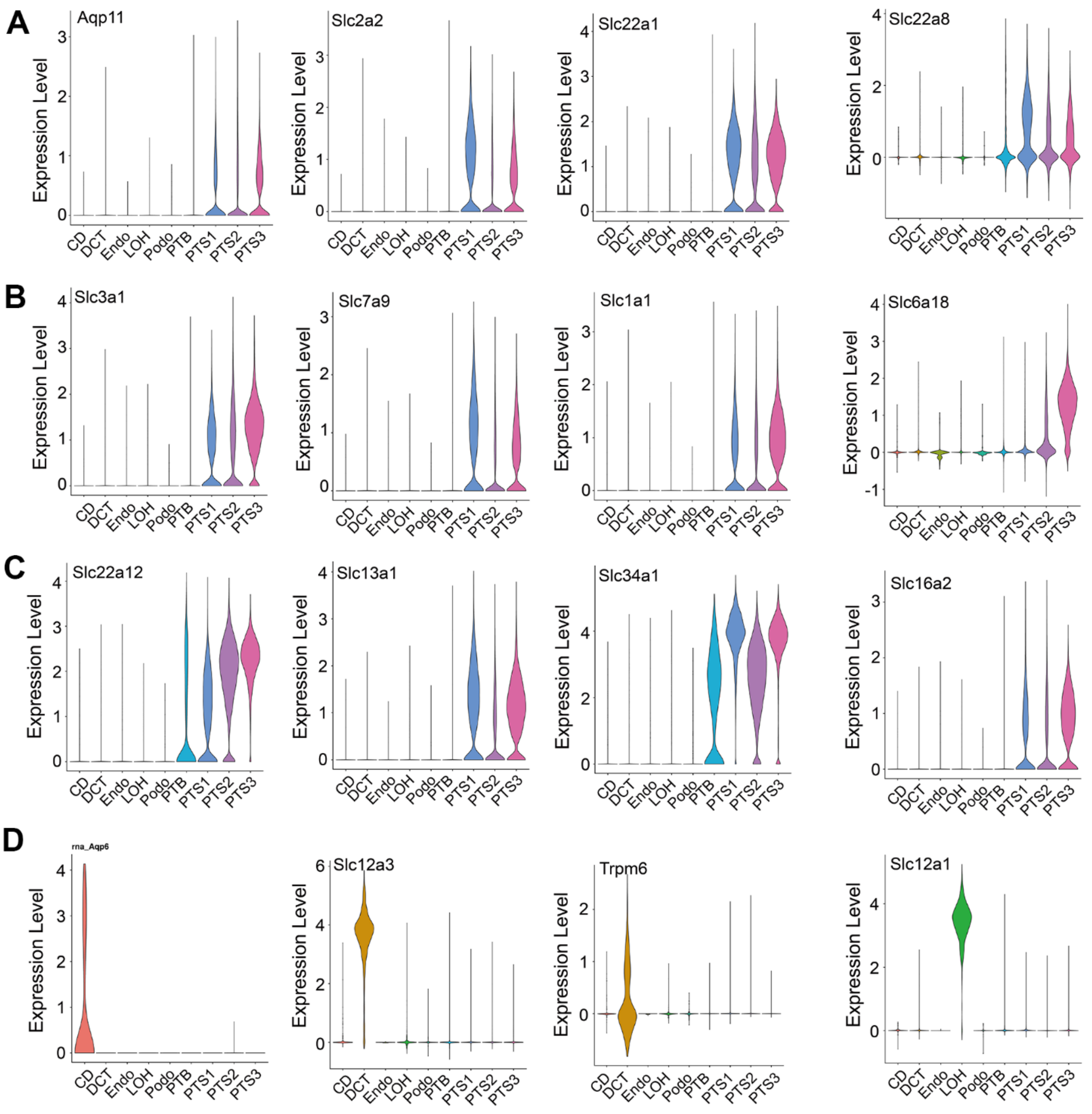

Supplementary Figure 1. Specific expression patterns of transporters along the renal tubule regions (PTB, PTS1, PTS2, and PTS3), collecting duct (CD), distal convoluted tubule (DCT), loop of Henle (LOH), podocytes (Podo), and endothelial cells (Endo). (**A**–**C**) Violin plots showing the transporters expressed specifically in proximal tubules. (**D**) Violin plots showing the transporters expressed specifically in the CD, DCT, and LOH.

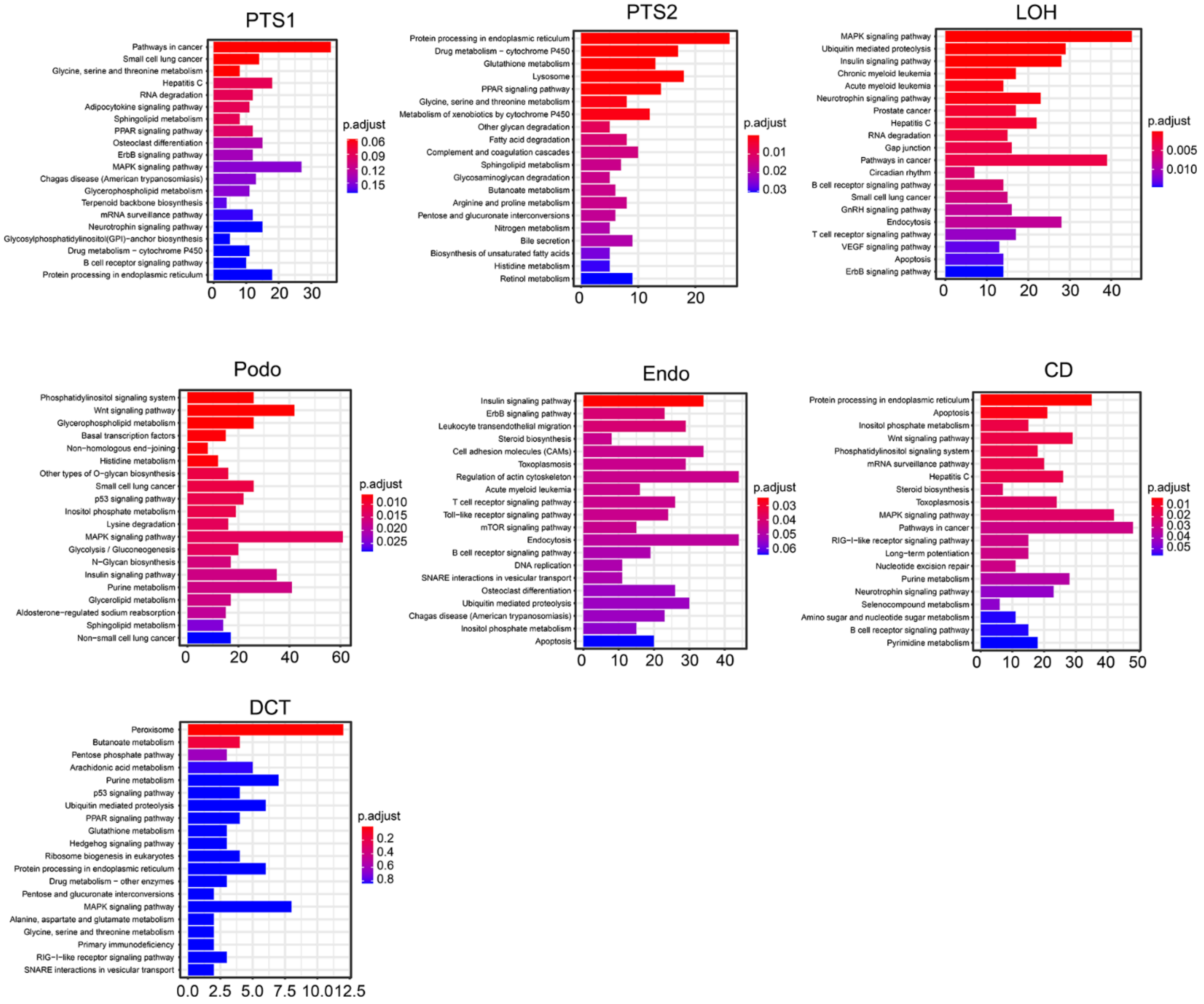

Supplementary Figure 2. Functional analysis of the upregulated genes in the PTS1, PTS2, LOH, Podo, Endo, CD, and DCT clusters using Kyoto Encyclopedia of Genes and Genomes pathway enrichment analysis. The color indicates corresponding significance values.

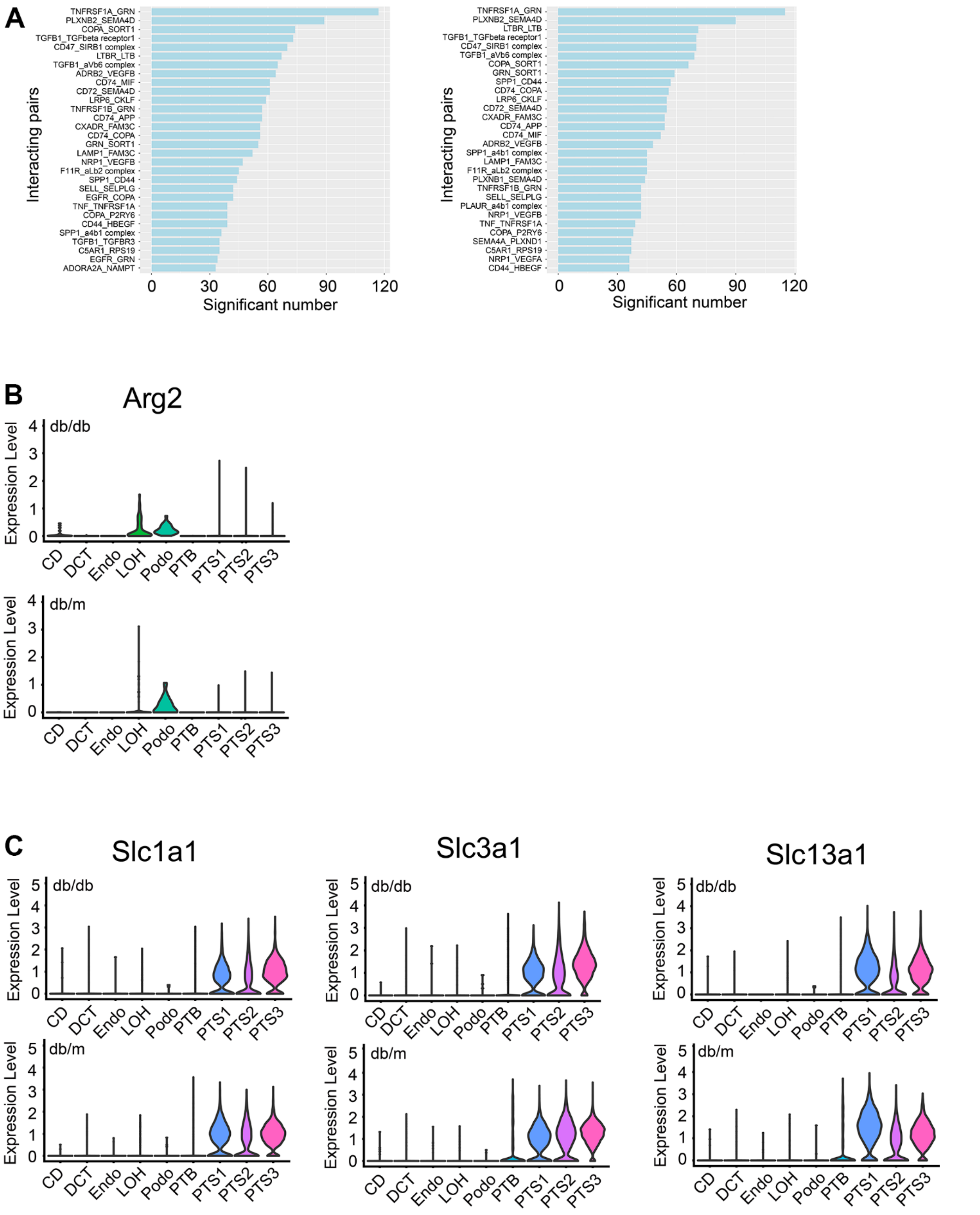

Supplementary Figure 3. Differential expression of key molecules and ligand receptors in db/db and db/m mice. (**A**) Bar plot showing the top 20 ligand–receptor pairs in db/m (left) and db/db (right) mice. (**B**) Violin plots showing the expression of *Arg2* in the kidneys of db/db and db/m mice. (**C**) Violin plots showing the expression of metabolic enzymes related to fatty acids in db/db and db/m mice.

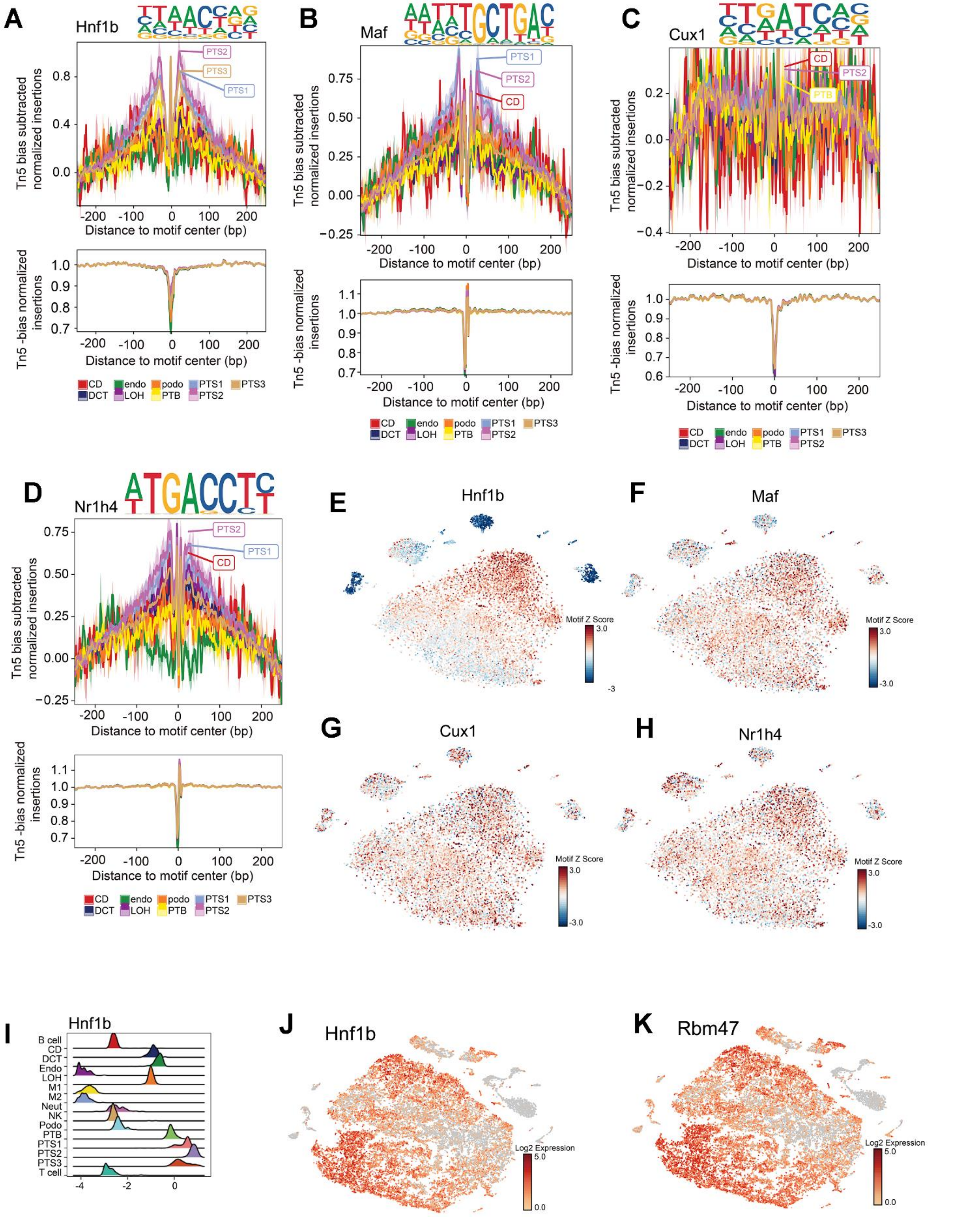

Supplementary Figure 4. Specific TF analysis in proximal tubule cells. (**A**–**D**) Footprinting of Hnf1b, Maf, Cux1, and Nr1h4. (**E**–**H**), the chromatin accessibility of the regions that Hnf1b, Maf, Cux1, and Nr1h4 bound were visualized using tSNE, with the accessibility level indicated by color intensity. (**I**) Differential motif enrichment analysis of Hnf1b; the density map shows the distribution of the motif deviation value. (**J**, **K**) Expression of Hnf1b and Rbm47 (the target genes of Hnf1b); the expression level is indicated by color intensity.

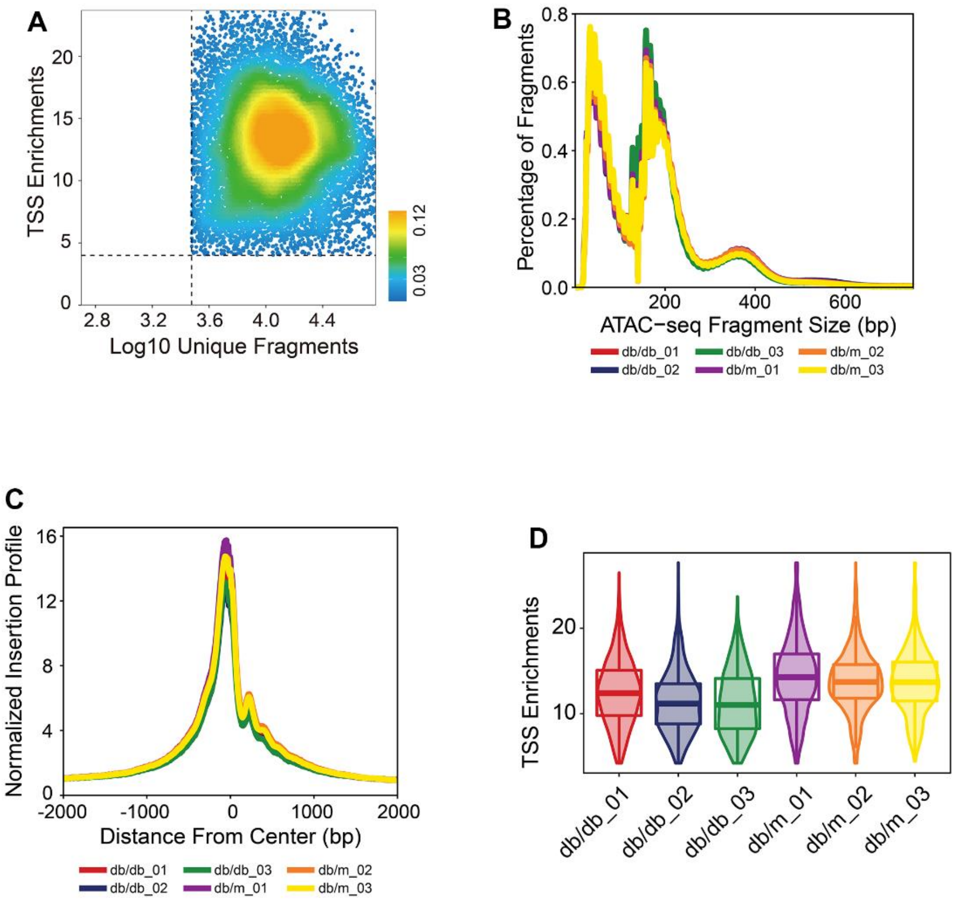

Supplementary Figure 5. Quality control (QC) of scATAC-seq data. Cells with a transcription start site (TSS) enrichment score > 4 and with > 3,000 fragments were screened. The results of six scATAC-seq samples after QC are shown. (**A**) Cell TSS score distribution after QC. (**B**) Insert size distribution showing periodic patterns. (**C**) TSS enrichment peak map. (**D**) Violin plot showing the TSS scores.
