# Supplementary material for: Analysis of potential biomarkers for diabetic kidney disease based on single-cell RNA-sequencing integrated with a single-cell sequencing assay for transposase-accessible chromatin
Source: Aging (Albany NY). 2023 Oct 11;15(19):10681–704. doi: 10.18632/aging.205107 (PMC10599739; doi:10.18632/aging.205107)
Supplement: Supplementary Table 1 [file aging-15-205107-s002.pdf]

SUPPLEMENTARY TABLES

Supplementary Table 1. Quality control.

	Estimated number of Cells	Mean reads per Cell	Median genes per cell	
db/db-01	6584	57271	1233	Before quality control
db/db-02	6845	57175	1474	Before quality control
db/db-03	8120	69249	1145	Before quality control
db/m-01	5463	64092	1500	Before quality control
db/m-02	9292	39063	2001	Before quality control
db/m-03	6529	61554	1817	Before quality control
	**cell.count**	**gene.median**	**UMI.mean**	
db/db-01	4141	2021	7918.96112	After quality control
db/db-02	4487	2426	10090.1306	After quality control
db/db-03	5278	1828	6765.928	After quality control
db/m-01	4136	1960	7814.31552	After quality control
db/m-02	8212	2242	8697.10935	After quality control
db/m-03	5804	2028	8379.16988	After quality control
