# Supplementary material for: Analysis of potential biomarkers for diabetic kidney disease based on single-cell RNA-sequencing integrated with a single-cell sequencing assay for transposase-accessible chromatin
Source: Aging (Albany NY). 2023 Oct 11;15(19):10681–704. doi: 10.18632/aging.205107 (PMC10599739; doi:10.18632/aging.205107)
Supplement: Supplementary Table 3 [file aging-15-205107-s004.pdf]

Supplementary Table 3. Cell number in each cluster.

	SR-db-db-01_number	SR-db-db-02_number	SR-db-db-03_number	SR-db-m-01_number	SR-db-m-02_number	SR-db-m-03_number	db/db toatl	db/db percent	db/m toatl	db/m percent
B cell	86	63	98	408	209	288	247	0.017762117	905	0.049856765
CD	46	16	18	16	27	27	80	0.005752912	70	0.003856324
DCT	194	106	193	122	150	169	493	0.035452323	441	0.024294844
endo	18	13	16	50	46	15	47	0.003379836	111	0.006115029
LOH	83	97	213	43	72	119	393	0.028261182	234	0.012891141
M1	90	48	68	122	127	126	206	0.014813749	375	0.020658881
M2	21	28	28	74	30	50	77	0.005537178	154	0.008483914
Neut	11	9	24	46	20	44	44	0.003164102	110	0.006059938
NK	10	5	25	119	37	68	40	0.002876456	224	0.012340238
podo	9	13	8	8	26	20	30	0.002157342	54	0.002974879
PTB	379	429	637	331	527	490	1445	0.10391198	1348	0.074261789
PTS1	1034	1151	1408	963	1590	1060	3593	0.258377679	3613	0.199041428
PTS2	1572	1579	1565	968	3501	2310	4716	0.339134187	6779	0.373457
PTS3	549	902	920	706	1798	918	2371	0.170501942	3422	0.188519
T cell	39	28	57	160	52	100	124	0.008917014	312	0.017188
