# Supplementary material for: Analysis of potential biomarkers for diabetic kidney disease based on single-cell RNA-sequencing integrated with a single-cell sequencing assay for transposase-accessible chromatin
Source: Aging (Albany NY). 2023 Oct 11;15(19):10681–704. doi: 10.18632/aging.205107 (PMC10599739; doi:10.18632/aging.205107)
Supplement: Supplementary Table 8 [file aging-15-205107-s009.pdf]

Supplementary Table 8. Baseline information of mice.

	Kidney weight (g)	Body weight (g)	Blood glucose level (mmol/L)
db/m-01	27.2	0.38	4.8
db/m-02	26.41	0.43	4.3
db/m-03	25.93	0.41	3.6
db/db-01	56.88	0.62	19.5
db/db-02	58.57	0.59	16.2
db/db-03	57.36	0.87	22.6
